# Magnetofection Enhances Adenoviral Vector-based Gene Delivery in Skeletal Muscle Cells

**DOI:** 10.4172/2157-7439.1000364

**Published:** 2016-04-05

**Authors:** Andrea Soledad Pereyra, Olga Mykhaylyk, Eugenia Falomir Lockhart, Jackson Richard Taylor, Osvaldo Delbono, Rodolfo Gustavo Goya, Christian Plank, Claudia Beatriz Hereñu

**Affiliations:** 1 Biochemistry Research Institute of La Plata (INIBIOLP)/National Scientific and Technical Research Council (CONICET), School of Medicine, National University of La Plata, La Plata, BA, Argentina (ZC 1900); 2 Ismaninger Street 22, Institute of Immunology and Experimental Klinikum rechts der Isar, Technical University of Munich, Munich, Germany (ZC 81675); 3 Department of Internal Medicine, Section on Gerontology and Geriatric Medicine, Wake Forest School of Medicine, Winston-Salem, NC, USA (ZC 27157); 4 IFEC-CONICET, Farmacology Department, School of Chemistry, National University of Cordoba, (ZC 5000) Córdoba, Argentina

**Keywords:** Gene delivery, Skeletal muscle, Magnetic nanoparticles, Adenoviral vectors, Magnetofection, Magneto-adenovectors

## Abstract

The goal of magnetic field-assisted gene transfer is to enhance internalization of exogenous nucleic acids by association with magnetic nanoparticles (MNPs). This technique named magnetofection is particularly useful in difficult-to-transfect cells. It is well known that human, mouse, and rat skeletal muscle cells suffer a maturation-dependent loss of susceptibility to Recombinant Adenoviral vector (RAd) uptake. In postnatal, fully differentiated myofibers, the expression of the primary Coxsackie and Adenoviral membrane receptor (CAR) is severely downregulated representing a main hurdle for the use of these vectors in gene transfer/therapy.

Here we demonstrate that assembling of Recombinant Adenoviral vectors with suitable iron oxide MNPs into magneto-adenovectors (RAd-MNP) and further exposure to a gradient magnetic field enables to efficiently overcome transduction resistance in skeletal muscle cells. Expression of Green Fluorescent Protein and Insulin-like Growth Factor 1 was significantly enhanced after magnetofection with RAd-MNPs complexes in C2C12 myotubes in vitro and mouse skeletal muscle in vivo when compared to transduction with naked virus. These results provide evidence that magnetofection, mainly due to its membrane-receptor independent mechanism, constitutes a simple and effective alternative to current methods for gene transfer into traditionally hard-to-transfect biological models.

## Introduction

Important advances have been made in gene transfer technology, with current efforts focusing on the design of safer and longer-lasting delivery vectors, as well as systems possessing cell-type specificity and regulatable expression. The association of viral vector-based gene delivery with nanotechnology now offers the possibility to develop more efficient gene transfer strategies for a number of applications *in vitro* and *in vivo* [1]. In 1978, Widder KJ introduced the concept of Magnetic Drug Targeting (MDT) and described how magnetically responsive therapeutic complexes could be concentrated in target areas of the body by means of external gradient magnetic fields [2]. Later, in the early 2000s this principle was applied to the delivery of nucleic acids into cells and a magnetic field-assisted transfection methodology emerged named magnetofection [3].

Magnetofection is based on the association of magnetic nanoparticles (MNPs) with non-viral or viral vectors in order to optimize nucleic acid delivery into biological systems in the presence of a magnetic field. The resulting “magnetic vectors” have to biocompatible enough for applications in living cells and organism and their magnetic response has to be sufficient to allow concentration at the target area under the influence of a magnetic force [1]. The current availability of stable, versatile and nontoxic MNPs offers the possibility to implement them for different biomedical pourposes such as anti-neoplasic therapy thru controlled hyperthermia, cell sorting and separation, drug delivery, gene and cell therapy and Magnetic Resonance Imaging [4]. Detailed protocols for MNPs synthesis and their biophysical characterization have been extensively described in the literature [5–7] as well as the mechanism for cellular uptake and metabolism [8].

One of the most important features of MNP-based transfection is the abilility to access virtualy any cell type due to its receptor-independent endocytic uptake. This is particularly useful in traditionaly hard-to-transfect and hard-to-transduce cells like NIH-3T3 fibroblasts [9], breast and mouse melanoma cancer cells [10], human peripheral blood lymphocytes [3] and mature skeletal muscle cells [11]. Skeletal muscle progenitors, called myoblasts, are mononucleated, mitoticaly active cells which divide rapidly until external cues signal them to exit the cell cycle and differentiate by fusing together into elongated, multinucleated myofibers (myotubes *in vitro*). It is well known that the skeletal muscle suffers a maturation-dependent loss of susceptibility to RAd uptake [12,13] which may represent a major constraint in the use of these vectors for gene transfer and therapy. It is believed that structural and biochemical changes are responsible for these decreased transduction efficiencies with adenoviral vectors. The entry of both wild and recombinant Adenovirus (Ad and RAd) into cells comprises a two-step sequence involving different membrane proteins: a high-affinity primary receptor, the Coxsackievirus and Adenovirus Receptor (CAR), that mediates the attachment of virus to the cell surface, and lower-affinity secondary receptors (αvβ3 and αvβ5 integrins) that allow internalization of the viral particles [14,15] Several studies have shown that in developing human, mouse and rat muscle, expression of the primary Ad membrane receptor CAR is severely downregulated even at early ages with CAR mRNA being barely detectable in adult myofibers [16]. Furthermore, it has been demonstrated that forced expression of CAR in myotubes by different aproches overcomes the poor RAd-mediated transducibility of these cells [17,18]. On the other hand, basal lamina and glycocalyx surrounding mature skeletal muscle cells appear to be an anatomical barrier that may limit the access of exogenously introduced virus [19].

In order to understand the physiology and physiopathology of the skeletal muscle, it is sometimes required to manipulate the levels or type of genes expressing this tissue. Adult skeletal muscle could be transfected *in vivo* with a low efficiency by either direct injection of naked plasmids [20], cationic lipids and neutral polymers [11,21] or most efficiently by electroporation [22] an established technology that transiently permeabilizes cell membrane with an electrical pulse, allowing the uptake of a wide spectrum of biological molecules-. None of these techniques, however, has the potential displayed by viral vectors, which are a highly effective transfection tools at the myoblast level [23,24].

The present work focuses on circumventing the low RAd-mediated transduction in differentiated skeletal muscle cells. Here we demonstrate the effective use of Magnetic nanoparticles-Recombinant Adenoviral vectors complexes (MNPs-RAd) to mediate transgene expression in mature C2C12 myotubes. We propose that MNPs-RAd represents a highly efficient method for transgene delivery in otherwise transfection-resistant differentiated skeletal muscle cells.

## Materials and Methods

### Myoblast and myotube cell cultures

C2C12 myoblasts (ATCC^®^ Number CRL-1772) were grown in high-glucose DMEM supplemented with 10% fetal calf serum (proliferation medium). When cells reached 70–80% confluence myoblasts were induced to differentiate and fuse into multinucleated myotubes by replacing the proliferation medium to DMEM with 2% donor horse serum (differentiation medium). After 6 days, multinucleated myotubes were infected for *in vitro* gene transfer protocol as described below. Chemicals were from Gibco^®^ (Thermofisher, Carlsbad, CA, USA) and Sigma-Aldrich^®^ (St Louis, MO, USA) and culture plates from Corning^®^ (New York City, NY, USA).

### Magnetic nanoparticles (MNPs) and magnetic field applicators

For formulation of the magnetic adenoviral complexes, the particles with a surface coating of 25-kDA branched polyethylenimine (PEI-25_Br_) combined with the fluorinated surfactant ZONYL FSA (lithium 3-[2-(perfluoroalkyl)ethylthio]propionate) were used, hereafter referred to as PEI-Mag2. [25] The aqueous MNP suspensions were sterilized using ^60^Co gamma-irradiation at a dose of 25 kGy. The MNP concentration was determined in terms of the iron content of the dry nanomaterial and the iron content in aqueous suspension of the stock nanomaterial as described previously [26]. The particles contain 560 mg Fe/g dry material weight. The mean iron oxide core size was calculated from the broadening of the X-ray diffraction peaks using the Scherrer formula. The static magnetic properties of the particles were evaluated by measuring the quasistatic magnetization in the applied DC-fields or M (H) by using the MPMS commercial susceptometer (Quantum Design, USA). Labeling of the PEI-Mag2 magnetic nanoparticles with a coating comprising PEI was performed using Atto550 NHS ester (Sigma-Aldrich^®^, Taufkirchen, Germany) in a 0.1 M Na-borate buffer, pH 8.5, followed by dialysis against water using a cassette dialysis device (Pierce Company, Dallas, Texas, USA) with a 3,500 MW cutoff as described in detail in [27]. A commercial magnetic plate (Oz Biosciences^®^, Marseille, France) was used to provide the proper magnetic field required for magnetic sedimentation of the magnetic vector upon *in vitro* magnetofection. It is composed of cylindrical-permanent- Nd-Fe-B magnets and generates a 0.3 T magnetic flux density and a gradient of 67–123T/m at a cell layer location. For *in vivo* magnetofection in C57BL/6 mice, a combination of two cylindrical magnets was used. After intramuscular administration of MNP-RAd-complexes, the magnets were positioned directly over the mouse hindlimb for 30 minutes, providing 430 mT of magnetic density at the surface of the magnet and a mean gradient of 18 mT/mm within the first centimeter.

### Recombinant adenoviral vectors RAd-IGF1 and RAd-GFP

A recombinant adenoviral vector (RAd) harboring the rat IGF1 gene (RAd-IGF1; IGF1 gene kindly donated by Dr. Peter Rotwein, Oregon Health Sciences University) was constructed in our laboratory by a variant of the 2-plasmid method [28] employing the AdMax plasmid kit (Microbix, Ontario, Canada). Briefly, the cDNA coding for the rat IGF1 gene (obtained from the mRNA for the IGF1b precursor form) [29] was excised from the plasmid pBluescript KS, subcloned in pCA14 and inserted into the multiple cloning site of shuttle pDC515, which contains an expression cassette consisting of the mouse cytomegalovirus promoter and the simian virus 40 polyadenylation signal, immediately upstream and downstream, respectively, of the multiple cloning site. The second plasmid from the kit, the genomic plasmid pBHGfrt(del)E1,3 FLP, consists of the entire genome of adenovirus 5, with deletions in the regions E1 and E3. In cotransfected HEK293 cells, FLP recombinase is readily expressed and efficiently catalyzes the site-directed recombination of the expression cassette of pDC515 into pBHGfrt (del) E1, 3 FLP, thus generating the genome of the desired vector RAd-IGF1. The newly generated RAd was rescued from HEK293 cell lysates and plaque purified. It was further purified by ultracentrifugation in CsCl gradient. Final virus stocks were titrated by a serial-dilution plaque assay. Another adenoviral vector, RAd-GFP, was constructed in our laboratory following the general procedures outlined above. It harbors a hybrid gene encoding the *Aequorea victoria* enhanced green fluorescent protein (GFP) driven by the mouse cytomegalovirus promoter. The vector was expanded in HEK293 cells and purified and titrated as for the RAd-IGF1 vector.

### Self-assembled magneto-adenoviral vectors and *in vitro* gene transfer

For in vitro transduction, MNP-to-virus ratios of 1.5 and 2.5 *femtogramms* of iron per Viral Physical Particle (VP), further referred to as *fg*Fe/VP, were tested based on previous data on optimization of the magnetic adenoviral vectors [5]. Previous experiments revealed that a multiplicity of infection (MOI) of 60 viral particles per cell yielded a good infection rate in this cell line and therefore was selected for the transduction protocols. Magnetic adenoviral vectors (RAd-PEI-Mag2) for *in vitro* transduction were assembled at MNP-to-virus ratios of 1.5 or 2.5 *fg*Fe/VP by mixing PEI-Mag2 nanoparticle suspension containing 11.25 or 18.75 g Fe/mL phosphate buffered saline (PBS), respectively, with equal volume of the adenovirus dilution containing 7.5 × 10^9^ physical virus particles per mL DMEM without additives. After incubation of the mixture for 20 minutes at room temperature to allow complex assembling the physic characteristics were measured. Myoblasts and myotubes were seeded on 24-well culture plates at a density of ≈1.2 × 10^5^ cells/well resulting in about 250000 cells per well 24 hours later upon infection. For transduction, 400 μl of the complexes (as prepared) were added to each well. As reference for later efficiency assessment, a naked virus dilution containing the same number of infective particles was used. Immediately after, the culture plate was positioned on top of the magnetic plate and exposed to the magnetic field for 30 min except for control cultures without magnet. After this incubation time, the magnetofection mixture was replaced with fresh cell culture medium.

### *In vivo* magnetofection-based gene transfer

*In vivo* magnetofection was performed in 10-month old C57BL/6 female mice. Magneto-adenovector RAd-Atto550PEI-Mag2 was prepared at MNP-to-virus ratio of 4 *fg*Fe/VP. For this, 9.8 μl of Atto550-PEI-Mag2 nanoparticle suspension containing 4.3 μg Fe were mixed with 0.2 μl of RAd-GFP adenovirus stock containing 10^9^ physical virus particles (10^7^ pfu) in sterile normal saline (0.9% NaCl). After incubation of the mixture for 20 minutes at room temperature to allow complex assembling, 10 μl of the complexes (as prepared) were injected into the Soleus muscle of mice as described below or diluted to 500 μL normal saline and used for complex characterization. Animals were anesthetized with a mixture of Ketamine/Xylazine (90/10 mg/kg of weight) by ip route and positioned ventrally on the operating table with both hindlimbs extended and gently immobilized. The hair that covers the posterior and external region of both legs was removed, the skin disinfected with topic povidone-iodine and finally a 1-cm long incision was made to access the posterior muscular compartment. After delicate dissection of the fascia and connective tissue, the left Soleus muscle was identified and injected, in its middle portion, with 10 ul of magnetic adenoviral vector using a (Hamilton^®^) syringe. The muscle fascia and skin were immediately sutured and the entire hind-limb was exposed to an external magnetic field for 30 minutes (see section 2.2. for magnet details). The right, contralateral Soleus muscle served as control and was injected only with naked RAd-GFP adenoviral vector (10^7^ pfu). Six days after magnetofection mice were euthanized by cervical dislocation. Muscles were promptly removed, fixed (4% PFA for 24 hours at 4°C), cryopreserved by sucrose gradient (0.25 M sucrose in PBS for 1 h, 0.5 M sucrose in PBS for 45 min, and finally 1.5 M sucrose in PBS for 30 min), embedded in tissue-freezing medium (Cryoplast^®^, Biopack, Buenos Aires, Argentina) and snap frozen in liquid nitrogen. Tissue blocks were stored at −80°C. Longitudinal 20-μm thickness sections were obtained and mounted with fluorescent mounting medium (Fluoromount G^™^, Electron Microscopy Sciences, PA, USA). Slides were analyzed using a fluorescence microscope as described on section 2.6. All experimental work involving laboratory animals was in compliance with local (Comité Institucional para el Cuidado y Uso de Animales de Laboratorio, FCM, UNLP) and international guidelines.

### Characterization of the magneto-adenovectors

The mean hydrodynamic diameter (Dh) and electrokinetic or ζ-potential of the MNPs and magnetic adenoviral complexes were determined by photon correlation spectroscopy (PCS) using a Malvern Zetasizer Nano Series 3000 HS (UK). Efficacy of the virus assembling with magnetic nanoparticles and stability of the complexes were assessed using iodine-127 labeled adenovirus as described in detail in ref. [5]. Briefly, Adenoviral particles were labeled with a iodine-127 as decribed were and assembled with PEI-Mag2 nanoparticles at different MNP-to-VP ratios in PBS for 30 min or further 1-to-1 diluted with Fetal Calf Serum, incubated for the next 30 and then exposed at the Magnetic Plate for 30 min, non-sedimented radioactivity was measured in a supernatant. To evaluate the rate of the magnetic vectors migration in applied gradient magnetic fields (magnetophoretic velocity) we have registered clarification kinetics of the suspensions by measuring space and time resolved extinction profiles using a customized (LUMiReader^®^) device equipped with a set of permanent magnets as described recently by Mykhaylyk et al. [30]. Concentration changes were detected by multiple extinction profiles taken at time intervals of 1 s with a spatial resolution of 30 μm at an analytical optical wavelength of 410 nm with Neodymium-Iron-Boron-Magnets positioned underneath the cuvette. From extinction profiles E is calculated averaged over a selected region of extinction profiles between meniscus and bottom of the cuvette and the relative extinction E/E0 was determined and plotted versus time, here E0 is an initial extinction of sample. In the linear range of Lambert-Beer law, the relative extinction E_rel_ can be considered as a relative concentration and represents the rate of the complexes remaining in suspension. The efficient magnetophoretic velocity of particles was calculated as υ=<L>/t, here <L> is a mean path of the particles upon magnetophoresis. With a sample hight of 8 mm (375 μl probe), <L>=4 mm. The relative concentration E/E0 plotted against the magnetophoretic velocity υ represents a cumulative distribution function Φ(υ), which describes the particle ratio having a magnetophoretic velocity υ_ι_ less or equal to the υ_ι_ value. The median values of magnetic moment M of the complex and number of magnetic nanoparticles *N=M/m_eff_* per complex/complex assembly were calculated as first described by Wilhelm et al. [31]. Here *m_eff_* =8.7 × 10–20 Am2 is an effective magnetic moment of the core of the nanoparticles accounting for the average diameter of the magnetite core of 9 nm and saturation magnetization of 62 emu/g iron.

### Fluorescence image acquisition

Cell culture images were obtained using an Olympus^®^ Digital Camera (E-330) attached to a Fluorescence Inverted Microscope (Olympus^®^ IX71) with a 20 X objective lens (Olympus^®^ Objective Lens LUCPLFLN 20XPH/NA 0.45). Tissue slides were analyzed with an Olympus Fluorescence Microscope (Olympus BX-51^®^; Olympus^®^ Objective Lens Plan and UPlanFL N) equipped with a DP70 CCD Video Camera (Olympus, Tokyo, Japan). The subsequent image processing was performed with (Image-Pro^®^ Plus) software (Version 5.1.2, MediaCybernetics, Inc).

### Quantitation of green fluorescent protein expression

Two days after transduction, the myoblasts and myotubes cultures were washed twice with PBS buffer and then incubated with 150 μl/ well of pre-cooled Lysis Buffer for 30 minutes at 4°C (0.1 % Triton X-100+EDTA 1 mM in PBS). The resulting lysates were spinned down at 13000 rpm during 2 minutes and the subsequent clean supernatants were aspirated and analyzed using a multimode microplate reader (Beckman Coulter, DTX 880 Multimode Detector) at excitation and emission wavelengths of 485/20 and 535/25 nm, respectively. To assure accurate comparison between gene delivery methods, unspecific fluorescence readings from cells alone, magnetic nanoparticles and lysis buffer were subtracted to the corresponding experimental group previous to data analysis. The results were expressed in Fluorescence Units.

### IGF1 measurements

In order to quantitate the production of IGF1, supernatant from myotube cultures was collected 48 hs after viral transduction with or without magnetofection and stored at −80°C until radioimmunoassay (RIA) was performed. IGF1 from samples was extracted by acid-ethanol cryo-precipitation [32]. The recombinant human IGF1 (rhIGF1, Chiron Corp., Emeryville, CA) was iodinated following the iodogen method [33]. The I^125^-labelled rhIGF1 was purified by exclusion chromatography on a PD-10 Sephadex G-25 M column (Cat. Num. 17-0851-01, GE Healthcare Bio-Sciences, PA, USA). The eluate corresponding to the rhIGF1 monomer was collected, the radioactivity was measured and then it was stored in aliquots at −20°C prior addition of glycerol. This material was used within 2 weeks of iodination and adjustments due to isotope decay were performed when necessary. The anti-rhIGF1 antibody (AFP4892898) was a gift from Dr A. F. Parlow (National Hormone and Peptide Program, Torrance, CA). Because the amino acid sequence of rat IGF1 differs from human IGF1 at only 3 of 70 residues in positions 20, 35, and 67 [34] is feasible to detect rIGF1 using this anti-human antibody [35]. Goat anti-rabbit antiserum (second antibody) was obtained with assistance from the School of Agricultural and Forestry Sciences, National University of La Plata.

### Transmission electronic microscopy image acquisition

Myoblasts and myotubes were seeded on 12-well culture plates (2.4 × 10^5^ cells/well). Viral transduction and magnetofection protocols were performed as described before. Once the incubation period was concluded, the RAd/RAd-MNPs mixtures were removed and the cell monolayer was fixed with 2% glutaraldehyde in phosphate buffer, (pH 7.2–7.4) for 2 h at 4°C. The cells were mechanically detached and spin at 1500 rpm for 10 minutes. The cellular pellets were re-fixed with 1% of osmium tetroxide for 1 h at 4ºC, dehydrated with ethanol and included in epoxy resin. Ultrathin slides (90 nm) were dye with uranyl acetate and lead-citrate and examined with a Transmission Electron Microscopy - JEM 1200 EX II (JEOL) (Electron Microscopy Core Lab, School of Veterinarian Medicine, National University of La Plata).

### Statistical analysis

For each experimental group at least three replicate samples were performed. Results are presented as Mean ± Standard Error of the Mean. Analysis of differences between Means was conducted by Student’s t-test and one-way analysis of variance (ANOVA), followed by Student-Newman-Keuls test (Sigma Plot 11.2, Systat Software, San Jose, CA). An alpha value of *p* ≤ 0.05 was considered statistically significant.

## Results

### Preparation and characterization of magnetic nanoparticles and magneto-adenovectors

The physicochemical properties of the MNPs used to formulate magnetic adenoviral complexes have been extensively described elsewhere [5,6]. PEI-Mag2 are core-shell nanoparticles with an iron oxide core with an average magnetite core size of 9 nm, saturation magnetization of 62 emu/g Fe and a surface coating consisting of the fluorinated surfactant ZONYL FSA (lithium 3-[2-(perfluoroalkyl) ethylthio] propionate) combined with 25-kDa branched polyethyleneimine. This results in a zeta potential of +55.4 ± 1.6 mV in water. The particles conjugated with Atto550 (Atto-550-PEI-Mag2) contain on average 15 molecules of the dye molecules per insulated magnetic nanoparticle providing red fluorescence. The loading with the dye was chosen to ensure bright fluorescence of the particle/complexes in the cell and to keep high enough positive surface charge to stabilize the particles and enable association of the particles with the negatively charged adenoviral particles into magnetic vectors.

**Figure 1:**
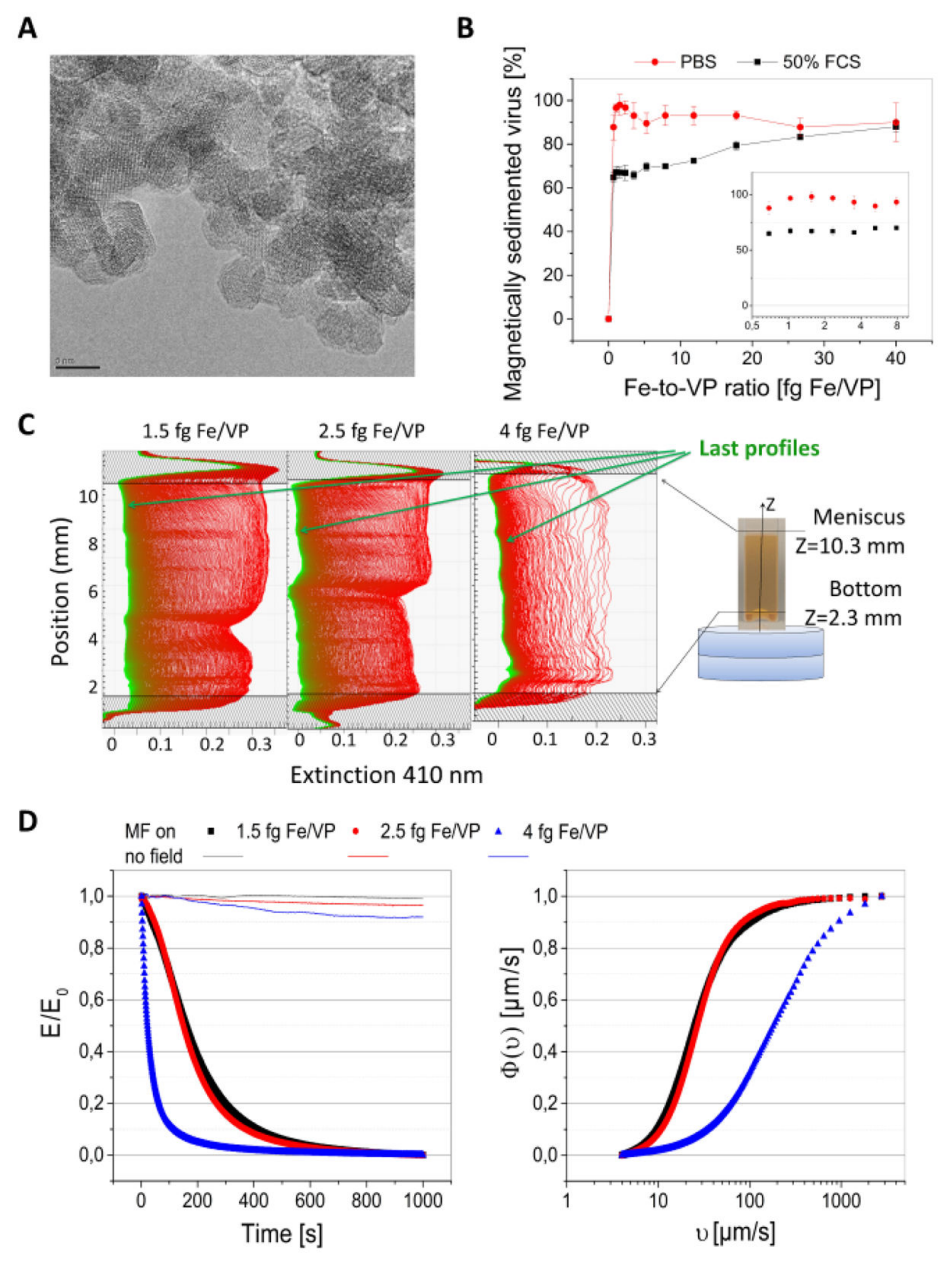
Magnetic nanoparticles and magnetic vectors: (A) Transmission Electron Microscopy image of the PEI-Mag2 nanoparticles, bar=5 nm. (B) Percent of the magnetically sedimented viral particles vs. MNP-to-Virus ratio (fg Fe/VP). Adenoviral particles labeled with a iodine-127 were assembled with PEI-Mag2 nanoparticles at different MNP-to-VP ratios in PBS for 30 min (curve “PBS”) or further 1-to-1 diluted with Fetal Calf Serum, incubated for the next 30 and then exposed at the Magnetic Plate for 30 min (curve “50%FCS”), non-sedimented radioactivity was measured in a supernatant. (C and D) Magnetophoretic mobility of the magnetic vectors characterized by kinetics of clearance of the suspensions in applied gradient magnetic fields. Figure (C) shows extinction profiles at 410 nm for the suspensions of the magnetic adenoviral complexes registered with a customized LUMiReader® device with 2 disk magnets positioned underneath optical cuvette at multiple points along the vertical axis of the cuvette (STEP-MAG measurements). Schematics at the right panel shows an optical cuvette and a set of Neodymium-Iron-Boron-Magnets positioned underneath the cuvette; resulting magnetic flux density and gradient averaged over vertical sample height were of 0.16 T and 33.5T/m, respectively. (D) Normalized integral Extinction at 410 nm, E/E_0_, averaged through the probe height versus time upon exposure to magnetic field (MF on) or with no filed applied (no field) and derived data on cumulative distribution functions of the effective magnetophoretic mobility F(u).

As shown in Figure 1, the amount of adenovirus that associated and magnetically sedimented with the PEI-Mag2 MNPs in PBS increased with increasing MNP-to-VP and amounted more than 90% of the initial virus particles at MNP-to-virus ratios of higher than 1 fg Fe/VP. When the formed complexes were incubated in 50% FCS for 30 min, partial destabilization of the adenovirus-MNP complexes occurred, however up to 60% of the adenovirus was still magnetically sedimented with PEI-Mag2 nanoparticles. Based on these results as well as on our previous data on optimization of the magnetic adenoviral vectors [5] the complexes for *in vitro* transduction were assembled at MNP-to-virus ratios of 1.5 and 2.5 fgFe/VP and for *in vivo* experiments a ratio of 4 fgFe per VP was used. The magneto-adenoviral vectors all possessed positive electrokinetic potential with a mean hydrodynamic diameter of 601, 521 and 937 nm, respectively (Table 1).

**Figure 2:**
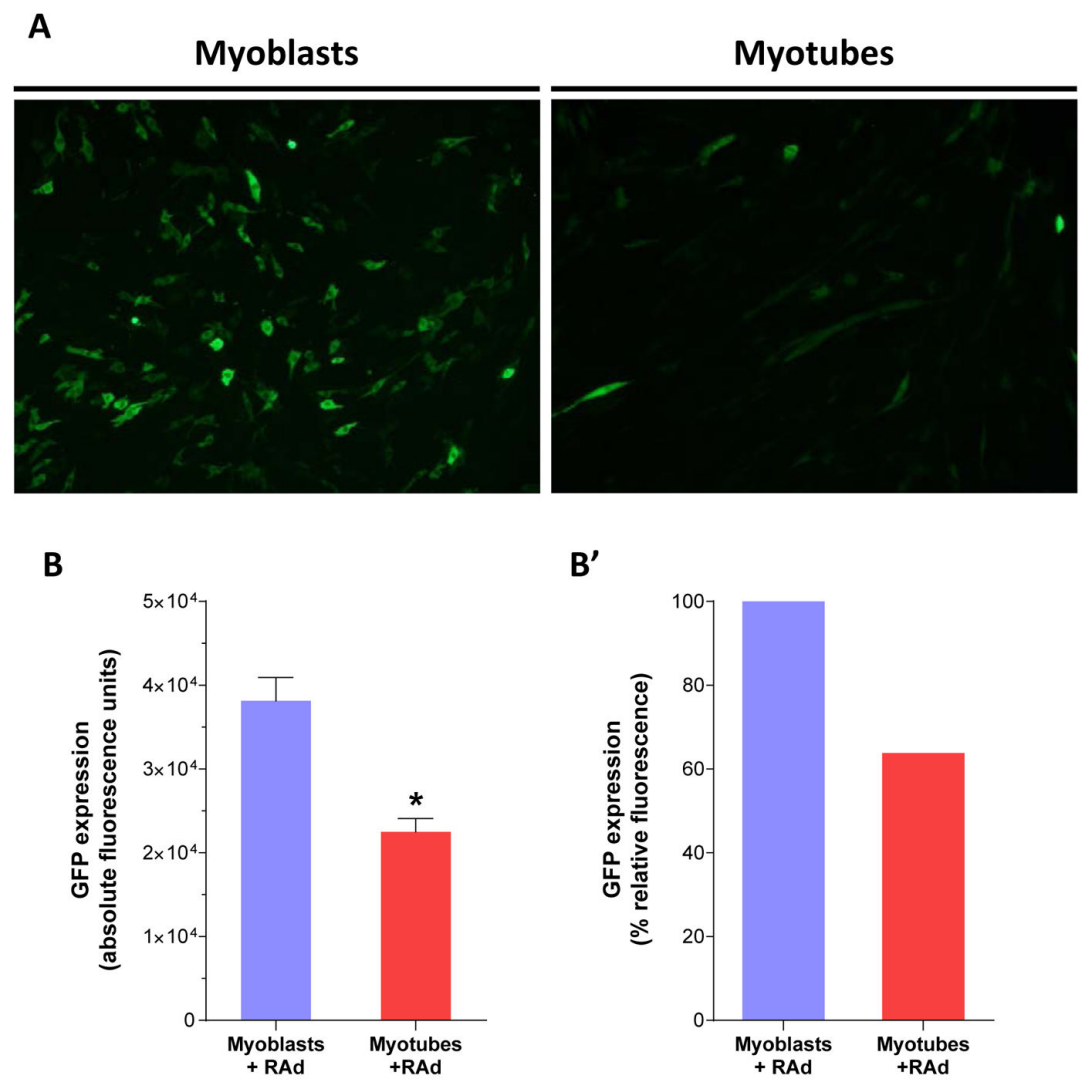
Conventional gene delivery with naked adenoviral vectors yields low efficiency in c2c12 myotubes: C2C12 myoblasts and fully differentiated myotubes were incubated for 60 minutes with a recombinant adenoviral vector, RAd-GFP, at a MOI of 60 viral particles per cell. To assess GFP expression, cell culture imaging and fluorescence quantitation were performed 48 hours after infection. A Representative microscopy images of myoblasts (left) and myotubes (right) transduced with RAd-GFP. After image acquisition, GFP quantitation was performed in cellular lysates. Comparison between both groups was plotted as absolute B and relative-to-myoblasts fluorescenceB’. Unspecific fluorescence readings from cells alone and lysis buffer were subtracted to each group previous to data analysis. * Statistically significant difference with p < 0.05 between the experimental groups. Data values represent mean ± S.E.M. n=3 for each group. Magnification, 10X.

The data on clarification of the complexes suspension when subjected to a defined gradient field (shown in terms of the relative extinction E/E_0_ at 410 nm plotted vs. time, Figure 1 left) were plotted against the magnetophoretic velocity υ (Figure 2 right) to give a cumulative velocity distribution function Φ(υ). As complexes did not show detectable sedimentation under only gravity as can be seen from the plots “no filed” in Figure 2 left, the Φ(υ) describes the particle ratio having a magnetophoretic velocity υ_ι_ less or equal to the υ_ι_ value. The derived median magnetophoretic values υ _0.5_ for the three viral complexes and the correspondent median values of magnetic moment M of the complex and number of magnetic nanoparticles *N* per complex/complex assembly are given in Table 1. The results suggest that there are multiple nanoparticles surrounding viral particles.

### Conventional gene delivery protocol with naked adenoviral vectors yields low efficiency in C2C12 myotubes

In order to determinate the transduction efficiency of C2C12 cell cultures by conventional Recombinant Adenoviral vector-based gene delivery, myoblasts and myotubes (6 days in differentiation medium) were incubated with RAd-GFP at a ratio of 60 infective viral particles per cell (MOI 60) and fluorescence microscopy images were taken 48 hours later. The number of transduced, GFP-positive myotubes (Figure 2, panel A2), as well as their fluorescence intensity, appeared lower when compared to images from myoblasts (Figure 2, panel A1).

Further quantitation of GFP levels was performed in cell lysates and expressed in absolute and relative Fluorescence Units (Figure 2B and B’). As expected, a significantly inferior (p<0.05) reading was obtained in transduced C2C12 myotubes compared to myoblasts at the same viral MOI (22487.7 ± 1599.2 and 38139.2 ± 2790.9 fluorescence units respectively), which stresses the need for new strategies to improve gene transfection into mature skeletal muscle.

### Magnetofection enhances viral vector-mediated gene delivery efficacy in C2C12 myoblasts and myotubes

**Figure 3:**
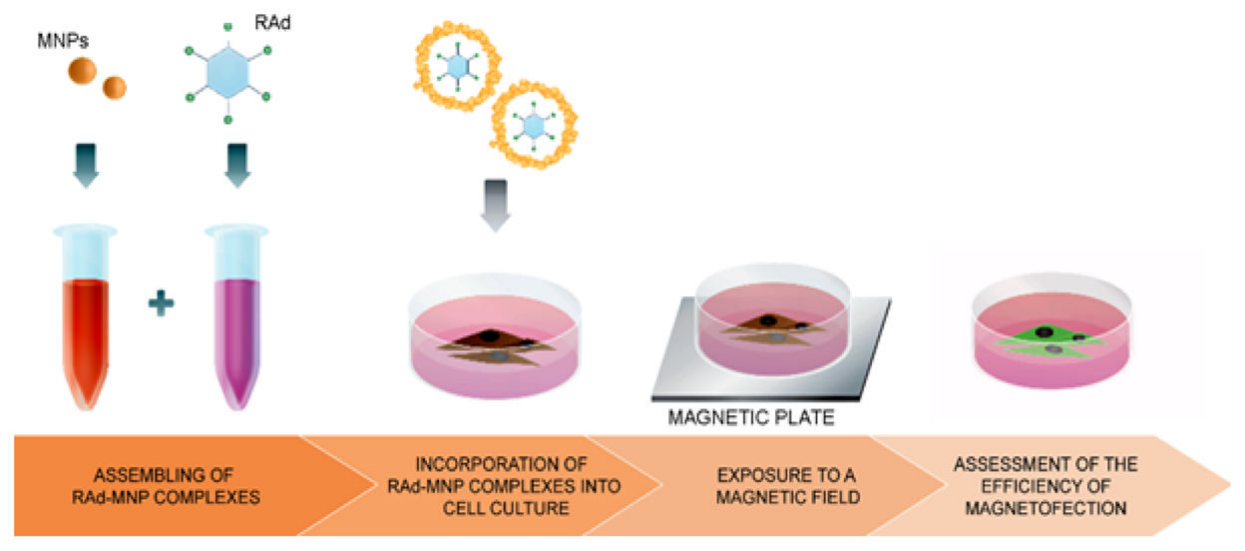
Magnetofection in cell culture: This illustration shows the general workflow for *in vitro* magnetofection. The RAd-MNP complexes are pre-assembled in the test tube and later introduced to the cell culture in a dropwise manner. The culture plate is then exposed to a magnetic field created by the magnetic plate placed under it and 48 hours later.

**Figure 4:**
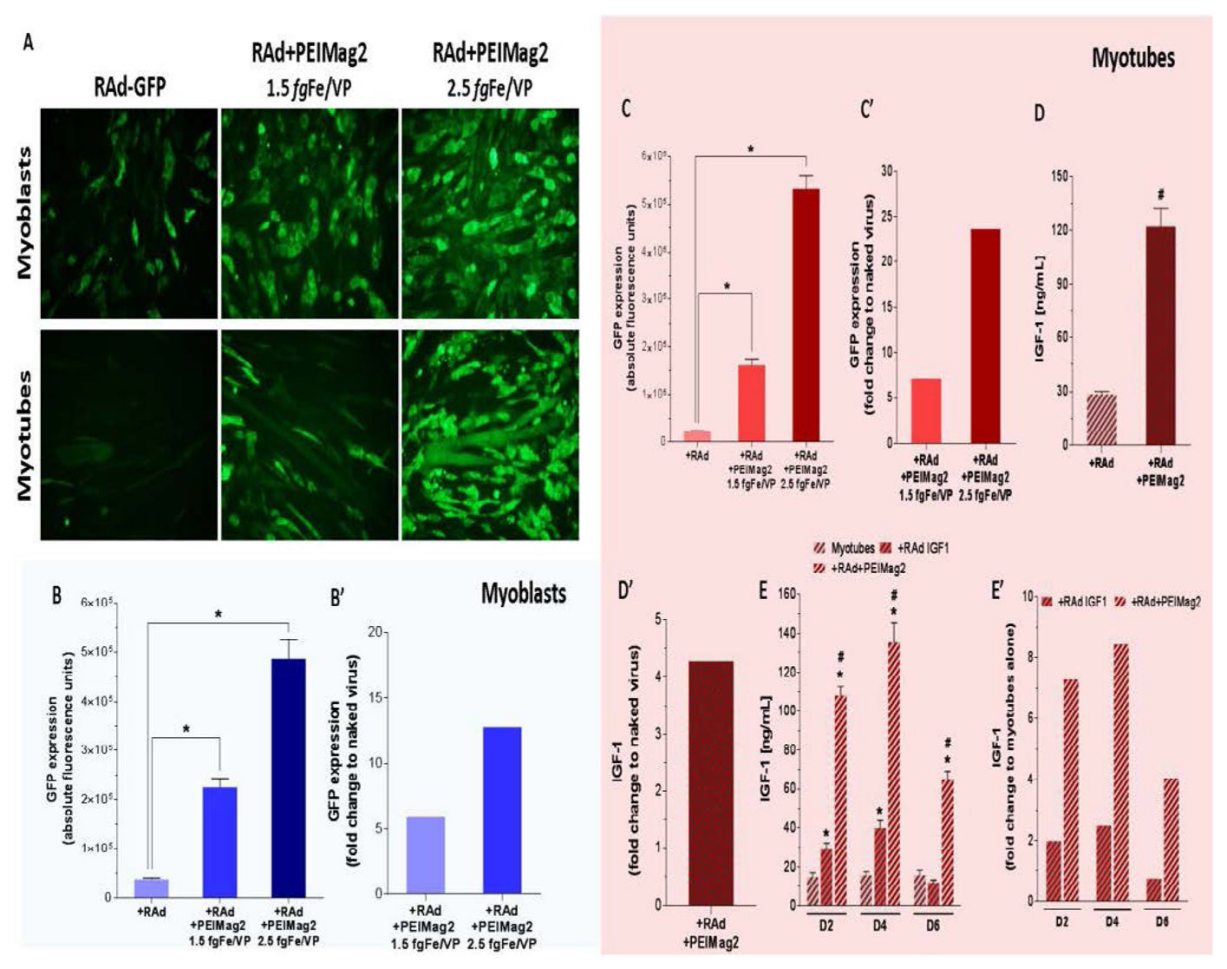
Magnetofection enhances viral vector-mediated gene delivery in C2C12 myoblasts and myotubes: C2C12 myoblasts and fully differentiated myotubes were incubated with either naked recombinant adenoviral vectors (RAd-GFP or RAd-IGF-1) or RAd+MNPs complexes (RAd+PEIMag2) and exposed to an optimal magnetic field for 30 minutes. To assess gene delivery efficiency, GFP expression was evaluated using microscopy imaging and fluorescence quantitation 48 hours after infection for both groups. In a similar manner, IGF-1 levels were measured in the culture medium by radioimmunoassay. A Representative microscopy images of myoblasts (upper panels) and myotubes (lower panels) infected with RAd-GFP and RAd-GFP+MNPs at 1.5 and 2.5 fgFe/VP MNP-to-Viral Particle ratios. After imaging, cells lysates were used to perform quantitation of GFP expression. Comparison between conventional viral transduction and magnetofection was plotted as absolute B and C and relative-to-naked virus fluorescence levels B’ and C’. Unspecific signal from cells alone, MNPs and lysis buffer were subtracted to each group previous to data analysis. To assess magnetofection efficacy with RAd-IGF-1, culture medium from myotubes was collected 48 hs after gene transfer protocols and processed for IGF-1 extraction and quantitation. Comparison between conventional viral transduction and magnetofection was plotted as absolute D and relative-to-naked virus concentration D’. IGF-1 basal levels in myotubes alone, as well as unspecific readings from extraction buffer, were subtracted to both experimental groups in order to show only viral vector-mediated IGF-1 production. In a subset of myotubes, IGF-1 expression was analyzed in the culture medium at days 2 (D2), 4 (D4) and 6 (D6) after gene delivery and compared between non-infected, transduced with naked virus and magnetofected cells. Plots show absolute E and relative-to-myotubes alone E’ IGF-1 levels for each time point. *Statistically significant difference with p < 0.05 between non-infected myotubes and naked virus or magnetofection groups. #Statistically significant difference with p < 0.05 between naked virus and magnetofection groups. Data values represent mean ± S.E.M. n=3 for GFP groups, n=4 for IGF-1 at 48 hs and n=6 for IGF-1 longitudinal analysis. fgFe/VP=femtograms of iron per Viral Particle

Here, we compared the efficacy of conventional RAd-based transduction protocol against magnetofection. Myoblasts and myotubes were incubated with either naked RAd-GFP or RAd-GFP+MNPs complexes as described before. PEI-Mag2 nanoparticles were chosen and tested at two different fgFe per Viral Particle (fgFe/VP) ratios. All cultures were exposed to the magnetic field for 30 minutes and additional incubation without magnet was performed for another 30-minute period. Afterwards, the transfection/magnetofection mixtures were removed (Figure 3). Images of transduced myoblasts (Figure 4, upper panel) and myotubes (Figure 4, lower panel,) were obtained 2 days later, and the quantitation of the fluorescence signal was subsequently performed. The use of RAd-MNPs complexes under the influence of a magnetic field achieved significantly higher transduction efficiency when compared to conventional protocol with naked RAd (p<0.05 for both experimental groups). For magnetofected myoblasts, the GFP levels were 5.9- and 12.8-fold higher with the use of 1.5 and 2.5 *fg*Fe/VP respectively (Figure 3B and B’). In the case of C2C12 myotubes, incubation with RAd-GFP+PEIMag2 complexes had a 7.1- (at 1.5 fgFe/VP) and 23.6-fold (at 2.5 fgFe/VP) enhancing effect (Figure 4C and C’).

Since skeletal muscle tissue is frequently used as an ectopic production site for several secretion molecules with paracrine and endocrine functions we decided to evaluate whether magnetofection could enhance IGF1 production and secretion *in vitro*. C2C12 myotubes were incubated with naked RAd-IGF1 or RAd-IGF1+PEI-Mag2 complexes as described before for RAd-GFP and 48 hours later IGF1 levels were measured by RIA in the cell culture supernatant. In the magnetofected myotubes, there was a 4.3-fold increase (p<0.001) in the secreted levels of IGF1 when compared to naked virus (Figure 4D and D’).

### Magnetofection achieves higher and more sustained levels of secreted IGF1 when compare to viral vectors alone

In a subset of C2C12 myotubes incubated with either RAd-IGF1 alone or RAd-IGF1+MNPs complexes, a short time course analysis of IGF1 expression was performed. Transduction efficiency was determined by measuring IGF1 levels in cell culture medium at days 2, 4 and 6 after gene delivery.

As shown in Figure 4E and E’, there was a significant initial overexpression of IGF1 peptide in both experimental groups when compared to basal levels from control, non-infected myotubes (1.98- and 7.27-foldfor naked RAd-IFG-1 and RAd+PEIMag2 respectively; *p<0.05). However, magnetofection was 3.3- to 5.9-fold more efficient (#p<0.05) than conventional naked RAd-mediated transduction at each time point. By day 6, secreted IGF1 from virally transduced myotubes had returned to basal levels while the concentration determined for the magnetofection-treated group remained considerably elevated (# p<0.05).

### The magnetic field is a major component of magnetofection-based gene delivery

Several literature reports suggested that MNPs could influence gene delivery even in the absence of a magnetic field (see Discussion for more details). To compare the efficacy of magnetofection with and without magnet the following experiment was performed.

**Figure 5:**
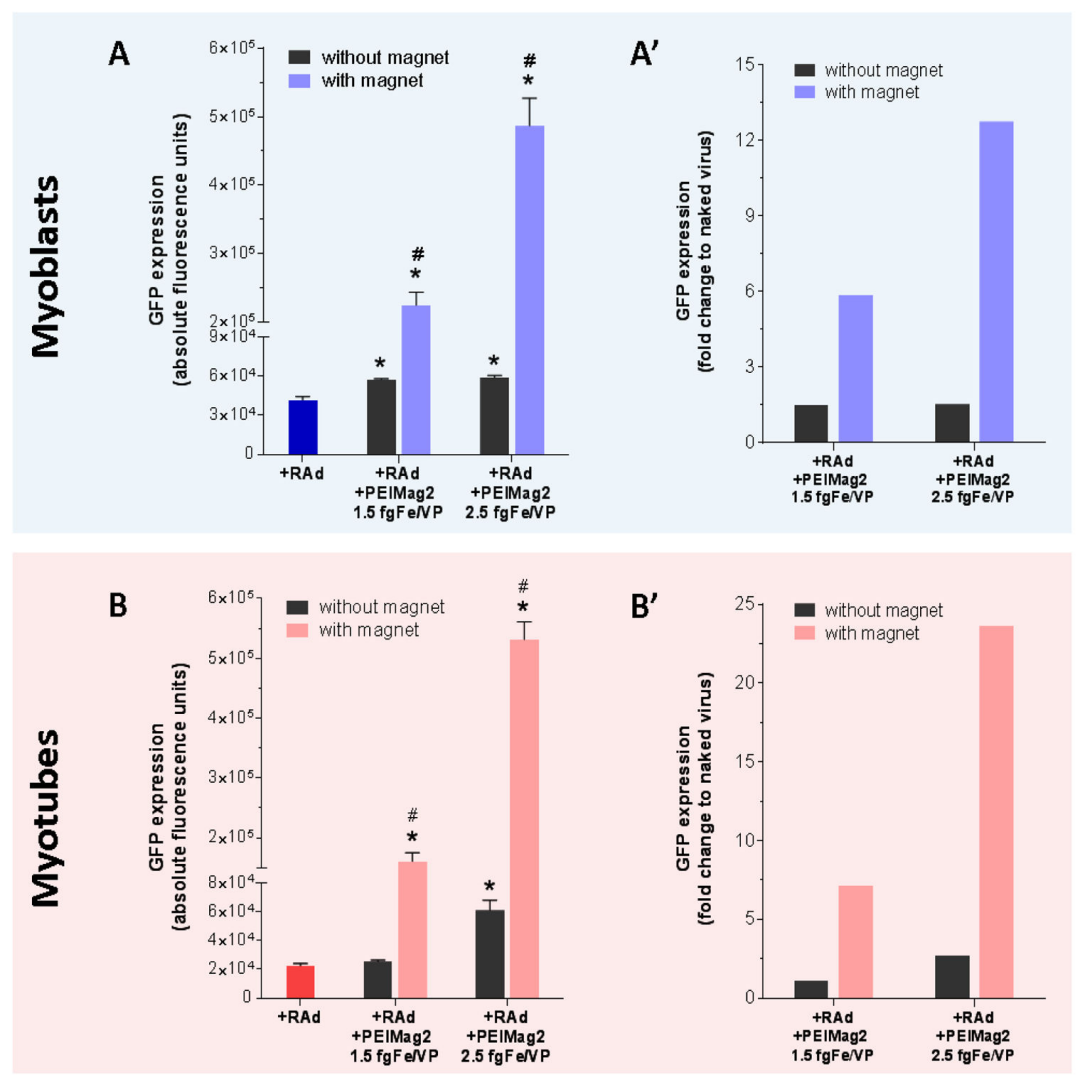
The magnetic field is a critical component of magnetofection-based gene delivery: C2C12 myoblasts and fully differentiated myotubes were incubated with either naked RAd or RAd+MagneticNanoParticles complexes and 48 hours later GFP expression was evaluated as described before. With and without magnetic field comparison was performed for both, myoblasts and myotubes. Results are plotted as absolute (A and B) and relative-to-naked virus fluorescence (A’ and B’). Unspecific fluorescence readings from cells alone, MNPs and lysis buffer were subtracted to each group previous data analysis. *Statistically significant difference with p < 0.05 between naked RAd and magnetofection with and without magnet. #Statistically significant difference with p < 0.05 between magnetofection with and without magnet. Data values represent mean ± S.E.M. n=3 for each group. fgFe/VP=femtograms of iron per Viral Particle

C2C12 myoblasts (Figure 5A and A’) and myotubes (Figure 5B and B’) were incubated with RAd-GFP+MNP complexes as described before and then placed over the magnetic plate for 30 minutes, except for the non-magnet group. A subset of cells were transduced only with naked RAd-GFP and served as reference group for comparison between techniques.

As predicted, a slightly but significant 1.13- to 2.71-fold increase in GFP expression was observed when the magneto-adenovectors were administer even in the absence of a magnetic field (*p<0.05). However, the magnetically-driven incorporation of RAd-MNP complexes into the cells substantially enhanced this effect when compared to no-magnet group (#p<0.05) and to naked virus (*p<0.05). Similar results were obtained with RAd-IGF1 (data not shown).

### Cellular uptake of RAd-MNPs complexes after magnetically-induced sedimentation

**Figure 6:**
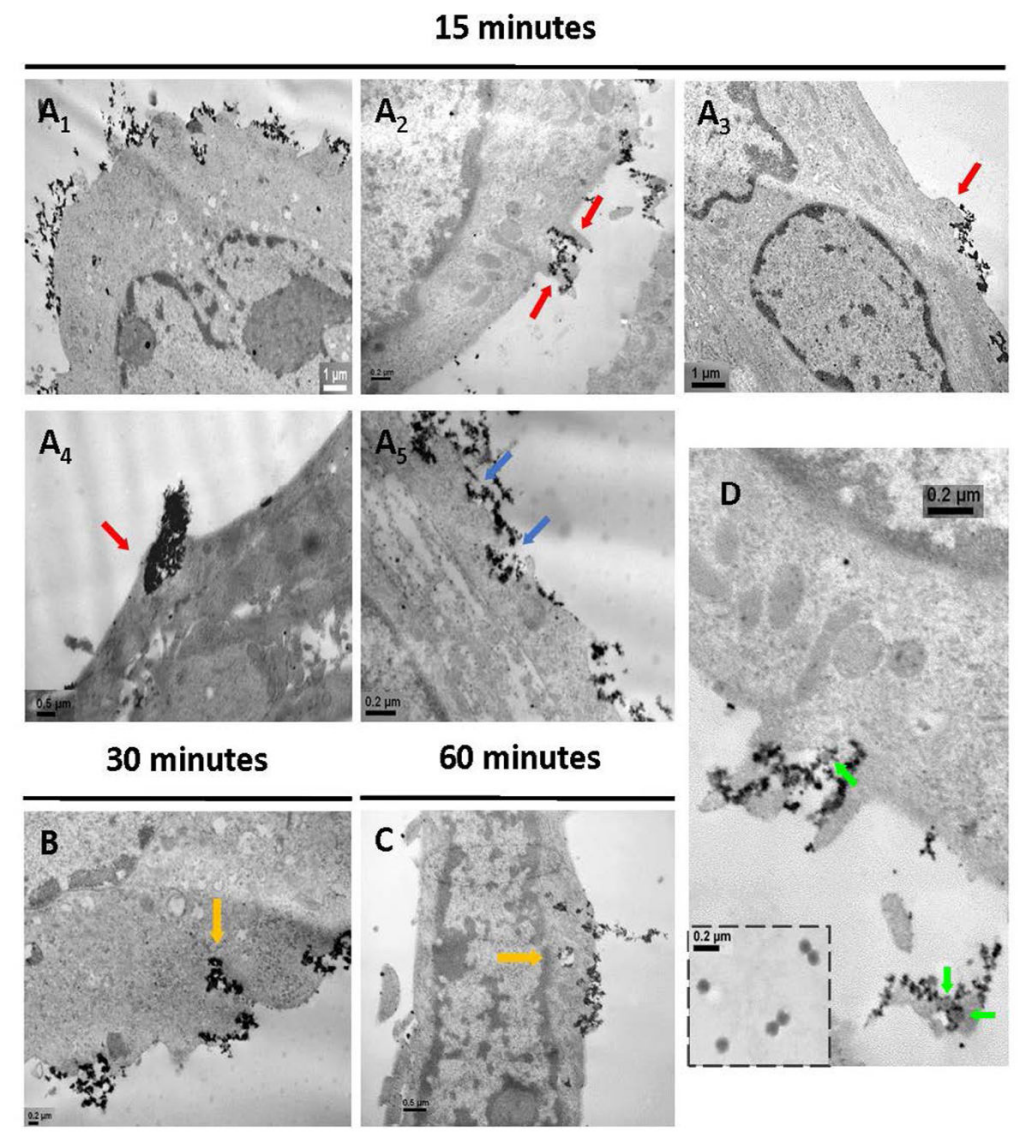
Cellular uptake of RAd-MNPs complexes after magnetically-induced sedimentation: Magnetofection protocol was applied to fully differentiated C2C12 myotubes and Transmission Electron Microscopy (TEM) images were taken at different time points after magnetic-field exposure. (A1) 15 minutes after magnetic-field exposure. Lower-magnification image to show the highly electron-dense (black) RAd-MNPs complexes distributed over the cell surface after magnetically-induced sedimentation. (A2–A5) Red arrows indicate membrane protrusions (lamellipodia-like structures) and blue arrows indicate membrane invaginations (pits). (B) After 30 minutes, engulfment and internalization of the RAd-MNPs complexes was evident (orange arrows). (C) 60 minutes after initial magnetic-field exposure, complexes could be visualized inside the cells, loaded into cytoplasmic vesicles (orange arrows). (D) Green arrows point to adenoviral particles surrounded by PEI-Mag2 magnetic nanoparticles while being engulfed by the myotube. Inset in the lower-right corner shows isolated Recombinant Adenoviral Vectors.

The uptake and intracellular trafficking of RAd-MNPs complexes were visualized at different time points using Transmission Electron Microscopy (TEM). The iron core of the PEI-Mag2 magnetic nanoparticles provides the sufficient electron-density to allow tracking without further processing or labeling of the sample. Within 15 minutes of exposure to the magnetic field, the magneto-adenovectors were scattered over the surface of C2C12 myotubes (Figure 6A_1_) and different engulfment processes seemed to become simultaneously activated. Several membrane protrusions (Figure 6A_2_–A_4_ red arrows) and invaginations (Figure 6A_5_, blue arrows) were already noticeable. At minute 30, endosome-like structures clearly started to form in the subcortical region of the cells (Figure 6B) and after 60 minutes, the RAd-MNPs complexes could be visualized inside the cells, loaded into cytoplasmic vesicles (Figure 6C). In some cases it was feasible to observe nearby the cell surface or attached to it, adenoviral particles surrounded by PEI-Mag2 nanoparticles (Figure 6D, green arrows).

**Figure 7:**
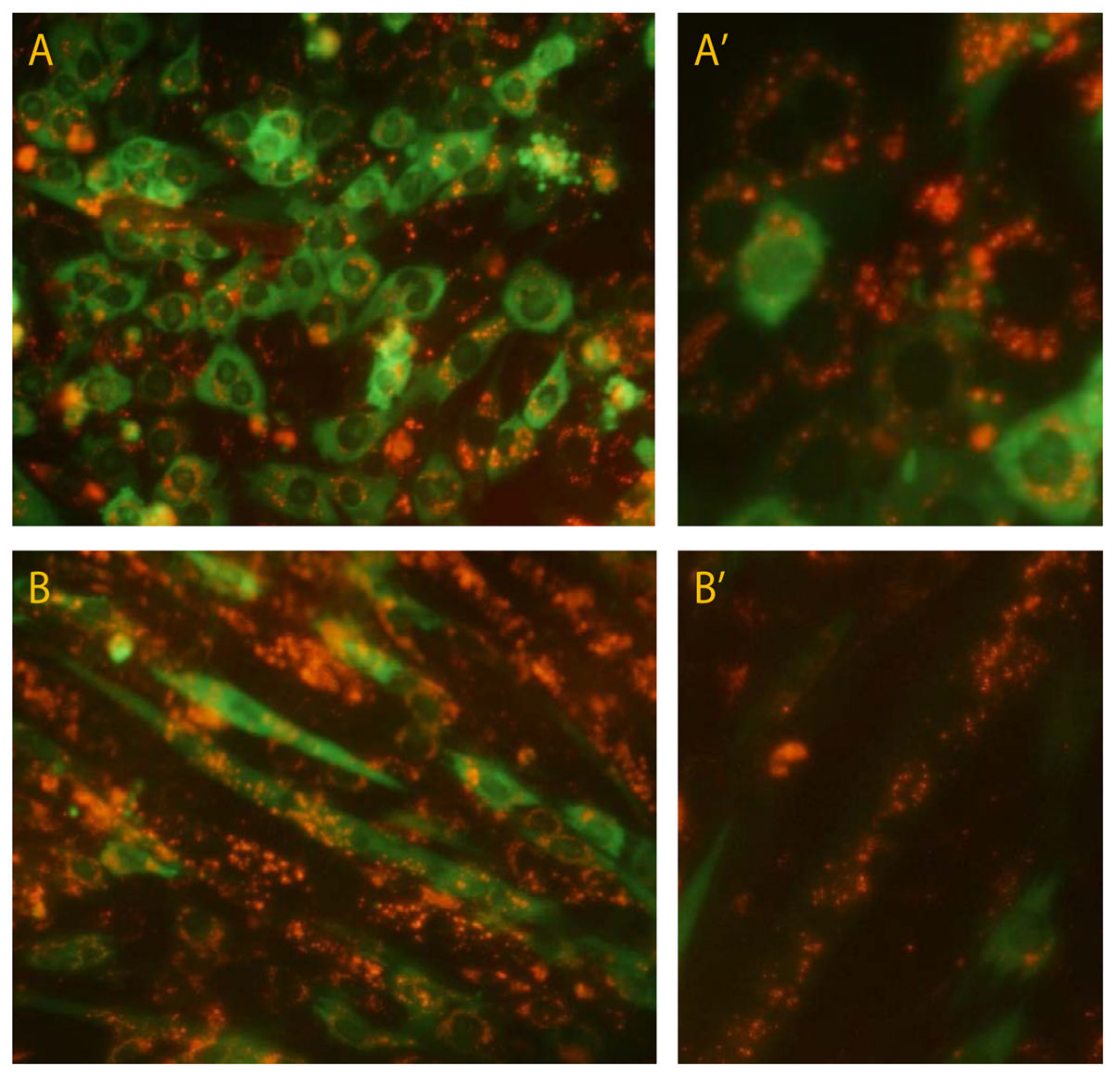
Intracellular fate and localization of RAd-MNPs complexes: To allow intracellular visualization and tracking, magnetoadenovectors were assembled with fluorescent MNPs and applied to C2C12 myoblasts and myotubes. Images were taken 48 hours afterwards. (A) simultaneous visualization of Green Fluorescent Protein expression (codified by the viral genome) and Atto550PEI-Mag2 nanoparticles (red fluorescence) in myoblasts. (A’) Enlarged area focused on the punctate, perinuclear localization of the red fluorescence. (B) Fully differentiated myotubes also displaying Atto550PEI-Mag2 localization around each nuclear domain. (B’) Enlarged picture of magnetofected myotubes.

The usage of magneto-adenovectors assembled with fluorescent MNPs (Atto500PEI-Mag2) allowed direct and simultaneous visualization of GFP expression (codified by the viral genome) and nanoparticle localization. As shown in Figure 7A and A’, 48 hours after magnetofection, green and red fluorescence colocalized within the same myoblasts. While GFP showed homogeneous cytoplasmatic distribution, signal from MNPs revealed a punctate and predominant perinuclear localization. Similar findings are reported here for C2C12 myotubes (Figure 7B and B’) were this intracellular disposition of Atto550PEI-Mag2 is clearly observed around each nuclear domain.

### *In vivo* magnetofection in mouse skeletal muscle

**Figure 8:**
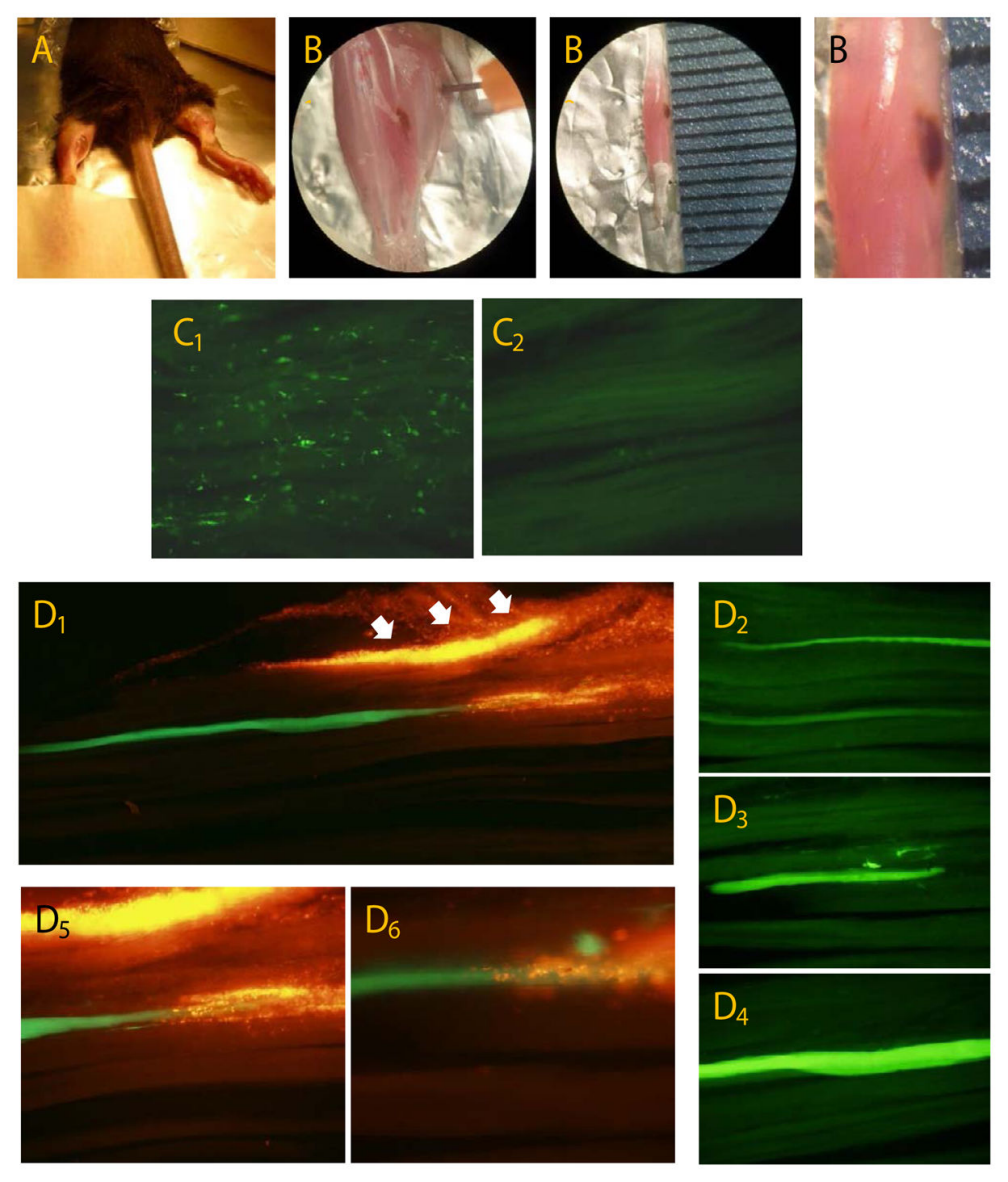
*In vivo* magnetofection in mouse skeletal muscle: (A) Our magnetofection protocol was evaluated in 10-month old C57BL/6 mice. Animals were anesthetized and positioned ventrally on the operating table with both hindlimbs extended and gently immobilized. Finally a 1-cm long incision was made to access the posterior muscular compartment. (B1, B2 and B3) Six days after magnetofection, freshly isolated Soleus revealed a brown area beneath the muscle surface where the RAd-MNPs were injected. (C1 and C2) Naked RAd-GFP resulted in transduction of only mononucleated cells with no GFP positive myofibers. (D1–D4) After magnetofection with RAd-MNPs complexes, GFP positive myofibers were noticeable and clearly differ from the surrounding tissue auto fluorescence. (D5 and D6) Co-localization of GFP expression and magnetic nanoparticles (Atto550PEI-Mag2 with red fluorescence) within the same myofiber.

Our magnetofection protocol was evaluated in adult C57BL/6 mice. As described before, a minor surgical procedure was performed in both hindlimbs in order to access the Soleus muscle and achieve direct intramuscular delivery (Figure 8A). The right hindlimb was transduced only with viral vectors and served as control whereas the left Soleus was treated with magneto-adenovectors and exposed to a magnetic field. Six days later mice were euthanized and muscles removed. Observation of the freshly isolated Soleus revealed a large, brown area beneath the muscle surface where the RAd-MNPs were injected and mobilized due to magnetic forces (Figure 8B1–B3). Fluorescence microscopy analysis of 20 μm-slides uncovered that injection of naked RAd-GFP resulted in transduction of only mononucleated cells with no Green Fluorescent Protein positive myofibers (Figure 8C1 and C2), similar to our *in vitro* results. The application of RAd-MNPs complexes driven by a magnetic field enhanced the transduction process allowing the entrance of RAd-GFP into the mature muscle fibers. Figure 8 (panels D1–D4) shows GFP+myofibers after being magnetofected. As discussed also in section 3.6., due to the fluorescent properties of Atto550-PEI-Mag2 nanoparticles (visualized in red), it was feasible to evaluate their distribution and co-localization with GFP inside the Soleus muscle (Figure 8D5 and D6). It is worth noticing that, although magneto-adenovectors were able to successfully transduce mature myofibers, many complexes remained clustered nearby the muscle surface (Figure 8D1, white arrows). This strengthens the need for further experimentation and improvement.

## Discussion

Magnetofection has been successfully applied *in vitro* to many biological systems and cell lines that are traditionally consider hard to transduce with adenoviral vectors like NIH-3T3 mouse fibroblasts and rat glioma cells [9], breast (MCF7) and mouse melanoma (B16F10) cancer cells [10], primary human peripheral blood lymphocytes (PBL16) [3] and human pancreatic carcinoma cells (EPP85-181RDB) [5]. Akiyama and collaborators [36] evaluated the expression of vascular endothelial growth factor (VEGF) in a monolayer of mouse C2C12 myoblasts using magnetofection with magnetite cationic liposomes (MCLs) coupled to a retroviral vector. This protocol increased transduction efficiency by approximately 7-fold compared with conventional retroviral method.

Despite these many studies, the effectiveness of magnetofection in fully differentiated, mature myotubes has not been evaluated heretofore. Our results demonstrate that conjugation of recombinant adenoviral vectors (RAd) with iron oxide based-magnetic nanoparticles (MNPs) can circumvent the low infection rate of myotubes and significantly enhance adenoviral vector-mediated gene delivery into mature muscle cells.

First, we described how transduction of fully differentiated C2C12 myotubes with naked RAd-GFP yield significantly lower transgene expression when compared to transduced myoblasts under the same conditions. This is in agreement with the well documented loss of the cell surface receptor CAR upon skeletal muscle maturation and differentiation [16].

Since magnetofection relies on membrane receptor-independent mechanisms for cell entry [3] we decided to test whether conjugation of RAd-GFP and RAd-IGF1 with poly-ethyleneimine-coated MNPs (PEIMag2) could improve gene transfer to skeletal muscle cells when exposed to a magnetic field. As mentioned before, the negative electrokinetic potential of adenoviral particles in aqueous media allows their assembly with cationic species of MNPs like PEI-coated due to electrostatically-induced aggregation. Available data on characterization of RAd-MNPs interaction showed that the efficiency of complex formation in the absence of serum depends mainly on the relative amounts of MNPs and viral particles (VP). Therefore, we selected for our experiments MNP-to-VP ratios of 1.5 and 2.5 *fg*Fe/ VP that were reported to achieve 75–80% of complex formation as measured by magnetically-driven sedimentation of the adenoviral particles [5].

RAd-GFP+PEIMag2-mediated infection of C2C12 myotubes in the presence of a magnetic field highly increased GFP expression up to 23-fold compared to conventional RAd-GFP procedure. These results also demonstrate that conjugation of RAd with MNPs has no deleterious effect upon the overall performance of the recombinant adenovirus as gene delivery vector. Similar results were obtained in C2C12 myoblasts were GFP levels were up to 13-fold higher.

Since skeletal muscle is often selected as an ectopic production site for secretion molecules with paracrine-endocrine functions [37] or antigenic properties (vaccines) we evaluated the potential of magnetofection as delivery method for IGF1 transgene into C2C12 myotubes. After incubation with RAd-IGF1+PEIMag2 and exposure to the magnetic field, the levels of secreted IGF1 growth factor into the culture medium were 4-fold higher than the ones achieved with naked RAd-IGF1. Considering that IGF1 synthesis and exocytosis are highly organized, energy-dependent processes [29] these results indirectly suggest that cell viability was not compromised during magnetofection.

It has been reported in the literature that MNPs can influence the cellular uptake of gene delivery vectors even in the absence of a magnetic field [5,38]. Here, we describe for C2C12 myoblasts and myotubes, a slightly but significant increase (1.13- to 2.71-fold) in transduction effectiveness of RAd-GFP+PEIMag2 complexes without magnet exposure over naked RAd-GFP. This could be explained by spontaneous, non-magnetically assisted sedimentation of magneto-adenovectors over the cell monolayer due to their larger size compared to viral particles alone. The accumulation of complexes in close contact with the plasmatic membrane triggered their internalization and further transgene expression. Nevertheless, comparison of no-magnet versus magnet groups demonstrated that the magnetic field is a major component of magnetofection since exposure to it significantly increased transduction of C2C12 muscle cells. This was probably due to more extensive sedimentation and enhanced cellular uptake of RAd-MNPs complexes.

Cellular uptake of nanoparticles (NPs) is mediated by different types of endocytosis taking place simultaneously within the same cell [8,39–41]. Selective blockage of the different internalization pathways in macrophage-like RAW264.7 cells revealed that dimercaptosuccinic acid-coated superparamagnetic iron oxide nanoparticles (DMSA-SPIONs) are internalized by clathrin- and caveolin-mediated endocytosis (CME) as well as macropinocytosis, a receptor-independent type of internalization [39]. Reports in C2C12 myoblasts showed that silica-based nanoparticles enter the cells mainly by CME and this correlated directly with high levels of transgene expression and therefore with overall transfection efficiency [40]. Similar results were obtained in human melanoma cells, human mesothelial cells and mouse fibroblasts exposed to silica-coated MNPs [38]. Internalization of PEI-coated MNPs, analogous to the ones used in our study, was thoroughly characterized in cultures of COS-7, SPC-A1, HeLa, HEP-G2 and BEAS-2B cells showing that they follow the same endocytic pathways [42,43]. Our TEM images from C2C12 myotubes incubated with RAd+PEIMag2 in the presence of a magnetic field showed perturbations of the cell surface that resemble internalization processes like the ones described above. Shortly after exposure to the magneto-adenovectors, protrusions of the cell plasma membrane to the external milieu like lamellipodia and ruffles and, at a lower extent, membrane invaginations (pits) started to form. These structures could be indicative of active macropinocytosis and CME. At a later time point (60 min), complexes could be visualized inside the cells, loaded into cytoplasmic vesicles, most likely to represent macropinosomes or early endosomes. It is worth noticing that, as reported in the literature [44], a correlation seemed to exist between the size of the RAd-MNPs aggregates and the internalization pathway, since macropinocytosis structures were more frequently observed engulfing large clusters.

We further analyzed the intracellular fate of magneto-adenovectors assembled with fluorescent MNPs (Atto500PEI-Mag2) which allowed us to simultaneously visualized GFP expression (codified by the viral genome) and nanoparticle localization. Forty-eight hours after magnetofection protocol, clusters of free MNPs and/or unassembled RAd-MNPs complexes displayed a predominant punctate pattern with perinuclear localization, consistent with the proposed internalization mechanisms and the classical centripetal distribution of internalized vesicles during the late stages of endosome-lysosome pathway. Similar findings were described in C2C12 myoblasts transfected with Rhodamine Red-labeled polyplexes that combine linear PEI substituted with histidine residues (His-PEI), were a perinuclear punctate pattern was observed after endocytosis [45]. Other formulations of NPs like magnetic polylactide-based coated with PEI [46], Boltorn-H30-co-(PEG10k)5-Fluorescent [47] poly(lactic-co-glycolic) acid-PEI-Hyaluronan [48] and magnetic polyacrylic acid-coated [49] also showed perinuclear localization after cellular uptake.

Despite the cell entry mechanism, any efficient gene delivery vector must be able to escape the endosome-lysosome pathway before degradation occurs. Moreover, those systems that do perform “endosomal escape” seem to achieve higher efficiency than direct cytosolic delivery (microinjection or electroporation) or formulations/ organisms unable to induce endosome-lysosome rupture [50,51]. In the case of wild type adenovirus and recombinant adenoviral vectors, interaction with the specific surface receptor (CAR) triggers clathrin-mediated endocytosis followed by endosomal escape, cytoplasmic transport and DNA import to the nucleus. Simultaneously, macropinocytosis is induced by the presence of viral particles in the cell surface, thus, enhancing viral uptake and release into the cytosol [52,53]. A similar scenario to avoid lysosome degradation has been described for many different nucleic acid-NPs complexes, especially PEI-coated formulations [54–56]. The “proton-sponge” theory [57] describes how polyamines, like PEI, can absorb free protons inside the endosomes/ lysosomes leading to further H+-ATPase mediated-accumulation and increase in the membrane potential past the equilibrium level. This triggers chloride anions and water diffusion which further increases the osmotic pressure and concomitant endosome/lysosome swelling and rupture. On the other hand, the acidic environment inside the degradative compartments favors the dissociation of negatively charged nucleic acids or adenoviral particles from the positively charged PEI-coated NPs allowing free DNA or RNA to reach the cytosol.

Recent reports suggest that DNA-NPs complexes that are able to access the cytoplasm can be uptake by autophagosomes, due to an autophagic response of the cell leading to final degradation or a “long-term” endosomal entrapment [58]. Although further experimentation is needed, we believe that this could be a possible scenario for our magneto-adenovectors as well. Re-capturing of free RAd-MNPs after endosome escape could explain the punctate perinuclear localization of fluorescent complexes 48 hours after the initial incubation mixture was removed and the occurrence of endosomal escape-autophagy uptake-autophagolysosome escape cycles could also contribute to a more prolonged presence of the RAd-MNPs complexes inside the cells, creating small “waves” of transgene release and expression, similar to a mini-pump system. We showed here that RAd-IGF1+PEIMag2 could achieve longer expression that naked RAd as shown by IGF1 levels measured at day 6 after gene delivery. Regardless of the exact mechanism that rules cell internalization and endosomal escape, we showed here that conjugation of recombinant adenovirus with PEIMag2 into magneto-adenovectors enhances viral gene delivery capability in hard-to-infect C2C12 myotubes, followed by an efficient transgene expression.

While *in vitro* magnetofection has become a highly successful technology for gene delivery in many different scenarios [1], *in vivo* applications still present themselves as a challenge. When applied to animal tissues, nanoparticle-based complexes encounter a significant set of barriers for penetration, mobility and distribution upon delivery [59]. Also, safety profile of NPs formulations needs to be in compliance with the requirements for biomedical usage. In the particular case of MNPs selection of an optimal external magnetic field is critical to produce the desired migration (magnetophoresis) and distribution of magneto-adenovectors within the tissue.

Despite many hurdles, magnetofection has been reported to enhance viral- or plasmid-mediated gene delivery into animal tissues like mouse brain [9,60,61], skeletal muscle [62], and liver tumors [63]. Here we describe how administration of magneto-adenoviral vectors followed by exposure to an external magnetic field was able to enhance transduction of soleus muscle in adult C57BL/6 mice. While naked RAd-GFP failed to transduce any muscle fiber, conjugation with Atto550PEI-Mag2 and subsequent exposure to an external magnetic field retrieved GFP+ myofibers 7 days later. Although magneto-adenovectors were injected in the mid-belly of Soleus muscle, after magnetophoresis took place most of the RAd-MNPs complexes were localized in a small area beneath the muscle surface in close proximity to the original position of the external magnet. This phenomena could be due to a high intensity magnetic field and/or prolonged exposure time that generated enhanced strong attraction across the tissue. Similar findings were reported after magnetofection in skeletal muscle [62] and in an orthotopic rat hepatocellular carcinoma model [63] suggesting that further improvement is needed to achieve a fair homogeneous distribution throughout the magnetofected organ.

## Conclusions

Development of reliable techniques for experimental manipulation of gene expression in mature skeletal muscle cells is critical to understand the molecular mechanisms involved in their physiology and pathophysiology. Magnetofection could serve as a powerful tool to aid Recombinant Adenoviral-mediated gene delivery into mature myofibers that are normally resistant to infection while preserving the benefits of these viral vectors. It is also worth noticing that other current uses of MNPs *in vivo* like cellular labeling, imaging modalities, drug delivery and magnetically induced hyperthermia [64] could benefit from any advances in the field.

## Figures and Tables

**Table 1:** Characteristics of the magnetic adenoviral vectors assembled at different MNP-to-VP ratios. After applying a magnetic flux density and field gradient of 0.16 T and 33.5 T/m, respectively, the following parameters were measured: electrokinetic or ξ**-**potential, intensity weighted mean hydrodynamic diameter *D_h_*, time for magnetic separation of 50% of the complexes t_0.5_, median values of the magnetophoretic mobility υ_0.5_, magnetic moment *M* of the complex and number of magnetic nanoparticles *N* per complex/complex assembly. *Meff=*effective magnetic moment of the core of the nanoparticles accounting for the average diameter of the magnetite core of 9 nm and saturation magnetization of 62 emu/g iron.

Complex	MNPs-to-VP ratio (fg Fe/VP)	ξ (mV)	D_h_(nm)	t_0.5 (s)_	_υ0.5_ (μm/s)	M (10^−15^ Am^2^)	N=M/m_eff_
RAd-PEI-Mag2	1.5	+11.4 ± 1.1	601	154	26	3.9	4.8E+04
RAd-PEI-Mag2	2.5	+10.2 ± 0.5	521	168	24	3.1	3.8E+04
RAd-Atto550-PEI-Mag2	4	+12.1 ± 0.2	937	22	184	43.2	5.2E+05

## References

[1] Plank C, Zelphati O, Mykhaylyk O (2011). Magnetically enhanced nucleic acid delivery. Ten years of magnetofection-progress and prospects. Adv Drug Deliv Rev.

[2] Widder KJ, Senyel AE, Scarpelli GD (1978). Magnetic microspheres: a model system of site specific drug delivery in vivo. Proc Soc Exp Biol Med.

[3] Scherer F, Anton M, Schillinger U, Henke J, Bergemann C (2002). Magnetofection: enhancing and targeting gene delivery by magnetic force in vitro and in vivo. Gene Ther.

[4] Gupta AK, Gupta M (2005). Synthesis and surface engineering of iron oxide nanoparticles for biomedical applications. Biomaterials.

[5] Tresilwised N, Pithayanukul P, Holm PS, Schillinger U, Plank C (2012). Effects of nanoparticle coatings on the activity of oncolytic adenovirus-magnetic nanoparticle complexes. Biomaterials.

[6] Mykhaylyk O, Zelphati O, Hammerschmid E, Anton M, Rosenecker J (2009). Recent advances in magnetofection and its potential to deliver siRNAs in vitro. Methods Mol Biol.

[7] Zhao X, Cui H, Chen W, Wang Y, Cui B (2014). Morphology, structure and function characterization of PEI modified magnetic nanoparticles gene delivery system. PLoS One.

[8] Oh N, Park JH (2014). Endocytosis and exocytosis of nanoparticles in mammalian cells. Int J Nanomedicine.

[9] Sapet C, Pellegrino C, Laurent N, Sicard F, Zelphati O (2012). Magnetic nanoparticles enhance adenovirus transduction in vitro and in vivo. Pharm Res.

[10] Choi JW, Park JW, Na Y, Jung SJ, Hwang JK (2015). Using a magnetic field to redirect an oncolytic adenovirus complexed with iron oxide augments gene therapy efficacy. Biomaterials.

[11] Lemieux P, Guérin N, Paradis G, Proulx R, Chistyakova L (2000). A combination of poloxamers increases gene expression of plasmid DNA in skeletal muscle. Gene Ther.

[12] Acsadi G, Jani A, Massie B, Simoneau M, Holland P (1994). A differential efficiency of adenovirus-mediated in vivo gene transfer into skeletal muscle cells of different maturity. Hum Mol Genet.

[13] Huard J, Lochmüller H, Acsadi G, Jani A, Holland P (1995). Differential short-term transduction efficiency of adult versus newborn mouse tissues by adenoviral recombinants. Exp Mol Pathol.

[14] Wickham TJ, Mathias P, Cheresh DA, Nemerow GR (1993). Integrins alpha v beta 3 and alpha v beta 5 promote adenovirus internalization but not virus attachment. Cell.

[15] Tomko RP, Xu R, Philipson L (1997). HCAR and MCAR: the human and mouse cellular receptors for subgroup C adenoviruses and group B coxsackieviruses. Proc Natl Acad Sci U S A.

[16] Nalbantoglu J, Pari G, Karpati G, Holland PC (1999). Expression of the primary coxsackie and adenovirus receptor is downregulated during skeletal muscle maturation and limits the efficacy of adenovirus-mediated gene delivery to muscle cells. Hum Gene Ther.

[17] Nalbantoglu J, Larochelle N, Wolf E, Karpati G, Lochmuller H (2001). Muscle-specific overexpression of the adenovirus primary receptor CAR overcomes low efficiency of gene transfer to mature skeletal muscle. J Virol.

[18] Kimura E, Maeda Y, Arima T, Nishida Y, Yamashita S (2001). Efficient repetitive gene delivery to skeletal muscle using recombinant adenovirus vector containing the Coxsackievirus and adenovirus receptor cDNA. Gene Ther.

[19] Cao B, Pruchnic R, Ikezawa M, Xiao X, Li J (2001). The role of receptors in the maturation-dependent adenoviral transduction of myofibers. Gene Ther.

[20] Wolff JA, Malone RW, Williams P, Chong W, Acsadi G (1990). Direct gene transfer into mouse muscle in vivo. Science.

[21] Gao X, Huang L (1995). Cationic liposome-mediated gene transfer. Gene Ther.

[22] Sandri M, Bortoloso E, Nori A, Volpe P (2003). Electrotransfer in differentiated myotubes: a novel, efficient procedure for functional gene transfer. Exp Cell Res.

[23] Wang Y, Fraefel C, Protasi F, Moore RA, Fessenden JD (2000). HSV-1 amplicon vectors are a highly efficient gene delivery system for skeletal muscle myoblasts and myotubes. Am J Physiol Cell Physiol.

[24] Sapru MK, McCormick KM, Thimmapaya B (2002). High-efficiency adenovirus-mediated in vivo gene transfer into neonatal and adult rodent skeletal muscle. J Neurosci Methods.

[25] Sanchez-Antequera Y, Mykhaylyk O, Thalhammer S, Plank C (2010). Gene delivery to jurkat t cells using non-viral vectors associated with magnetic nanoparticles. International Journal of Biomedical Nanoscience and Nanotechnology.

[26] Mykhaylyk O, Antequera YS, Vlaskou D, Plank C (2007). Generation of magnetic nonviral gene transfer agents and magnetofection in vitro. Nat Protoc.

[27] Vlaskou D, Plank C, Mykhaylyk O (2013). Magnetic and acoustically active microbubbles loaded with nucleic acids for gene delivery. Methods Mol Biol.

[28] Bett AJ, Haddara W, Prevec L, Graham FL (1994). An efficient and flexible system for construction of adenovirus vectors with insertions or deletions in early regions 1 and 3. Proceedings of the National Academy of Sciences of the United States of America.

[29] Daughaday WH, Rotwein P (1989). Insulin-like growth factors I and II. Peptide, messenger ribonucleic acid and gene structures, serum, and tissue concentrations. Endocr Rev.

[30] Mykhaylyk OLD, Vlaskou D, Schoemig V, Detloff T, Krause D (2015). Magnetophoretic mobility: Determination by space and time resolved extinction profiles (in-situ visualization).

[31] Wilhelm C, Gazeau F, Bacri JC (2002). Magnetophoresis and ferromagnetic resonance of magnetically labeled cells. Eur Biophys J.

[32] Breier BH, Gallaher BW, Gluckman PD (1991). Radioimmunoassay for insulin-like growth factor-I: solutions to some potential problems and pitfalls. J Endocrinol.

[33] Fraker PJ, Speck JC (1978). Protein and cell membrane iodinations with a sparingly soluble chloroamide 3,4,6-tetrachloro-3a,6a-diphrenylglycoluril. Biochemical and biophysical research communications.

[34] Shimatsu A, Rotwein P (1987). Mosaic evolution of the insulin-like growth factors. Organization, sequence, and expression of the rat insulin-like growth factor I gene. J Biol Chem.

[35] Lee PD, Baker BK, Liu F, Kwan EY, Hintz RL (1996). A homologous radioimmunoassay for rat insulin-like growth factor-I (IGF-I): implications for studies of human IGF-I physiology. J Clin Endocrinol Metab.

[36] Akiyama H, Ito A, Kawabe Y, Kamihira M (2010). Genetically engineered angiogenic cell sheets using magnetic force-based gene delivery and tissue fabrication techniques. Biomaterials.

[37] Goldspink G (2003). Skeletal muscle as an artificial endocrine tissue. Best practice and research Clinical endocrinology & metabolism.

[38] Prijic S, Scancar J, Romih R, Cemazar M, Bregar VB (2010). Increased cellular uptake of biocompatible superparamagnetic iron oxide nanoparticles into malignant cells by an external magnetic field. J Membr Biol.

[39] Gu J, Xu H, Han Y, Dai W, Hao W (2011). The internalization pathway, metabolic fate and biological effect of superparamagnetic iron oxide nanoparticles in the macrophage-like raw 264.7 cell. Science China Life sciences.

[40] Poussard S, Decossas M, Le Bihan O, Mornet S, Naudin G (2015). Internalization and fate of silica nanoparticles in C2C12 skeletal muscle cells: evidence of a beneficial effect on myoblast fusion. Int J Nanomedicine.

[41] Zhang S, Gao H, Bao G (2015). Physical Principles of Nanoparticle Cellular Endocytosis. ACS Nano.

[42] Ma Y, Zhang Z, Wang X, Xia W, Gu H (2011). Insights into the mechanism of magnetofection using MNPs-PEI/pDNA/free PEI magnetofectins. Int J Pharm.

[43] Huth S, Lausier J, Gersting SW, Rudolph C, Plank C (2004). Insights into the mechanism of magnetofection using PEI-based magnetofectins for gene transfer. J Gene Med.

[44] Akinc A, Battaglia G (2013). Exploiting endocytosis for nanomedicines. Cold Spring Harb Perspect Biol.

[45] Billiet L, Gomez JP, Berchel M, Jaffrès PA, Le Gall T (2012). Gene transfer by chemical vectors, and endocytosis routes of polyplexes, lipoplexes and lipopolyplexes in a myoblast cell line. Biomaterials.

[46] Chorny M, Polyak B, Alferiev IS, Walsh K, Friedman G (2007). Magnetically driven plasmid DNA delivery with biodegradable polymeric nanoparticles. FASEB journal: official publication of the Federation of American Societies for Experimental Biology.

[47] Zeng X, Zhang Y, Nyström AM (2012). Endocytic uptake and intracellular trafficking of bis-MPA-based hyperbranched copolymer micelles in breast cancer cells. Biomacromolecules.

[48] Maiolino S, Russo A, Pagliara V, Conte C, Ungaro F (2015). Biodegradable nanoparticles sequentially decorated with Polyethyleneimine and Hyaluronan for the targeted delivery of docetaxel to airway cancer cells. J Nanobiotechnology.

[49] Bregar VB, Lojk J, Suštar V, Veranič P, Pavlin M (2013). Visualization of internalization of functionalized cobalt ferrite nanoparticles and their intracellular fate. Int J Nanomedicine.

[50] Kloeckner J, Boeckle S, Persson D, Roedl W, Ogris M (2006). DNA polyplexes based on degradable oligoethylenimine-derivatives: Combination with egf receptor targeting and endosomal release functions. Journal of controlled release: official journal of the Controlled Release Society.

[51] Chang KL, Higuchi Y, Kawakami S, Yamashita F, Hashida M (2010). Efficient gene transfection by histidine-modified chitosan through enhancement of endosomal escape. Bioconjug Chem.

[52] Meier O, Boucke K, Hammer SV, Keller S, Stidwill RP (2002). Adenovirus triggers macropinocytosis and endosomal leakage together with its clathrin-mediated uptake. J Cell Biol.

[53] Meier O, Greber UF (2004). Adenovirus endocytosis. J Gene Med.

[54] Bakhru SH, Altiok E, Highley C, Delubac D, Suhan J (2012). Enhanced cellular uptake and long-term retention of chitosan-modified iron-oxide nanoparticles for MRI-based cell tracking. Int J Nanomedicine.

[55] Gujrati M, Malamas A, Shin T, Jin E, Sun Y (2014). Multifunctional cationic lipid-based nanoparticles facilitate endosomal escape and reduction-triggered cytosolic siRNA release. Mol Pharm.

[56] Wang F, Zhang W, Shen Y, Huang Q, Zhou D (2015). Efficient RNA delivery by integrin-targeted glutathione responsive polyethyleneimine capped gold nanorods. Acta Biomater.

[57] Behr J-P (1997). The proton sponge: A trick to enter cells the viruses did not exploit. CHIMIA International Journal for Chemistry.

[58] Remaut K, Oorschot V, Braeckmans K, Klumperman J, De Smedt SC (2014). Lysosomal capturing of cytoplasmic injected nanoparticles by autophagy: An additional barrier to non viral gene delivery. Journal of controlled release: official journal of the Controlled Release Society.

[59] Lane LA, Qian X, Smith AM, Nie S (2015). Physical chemistry of nanomedicine: understanding the complex behaviors of nanoparticles in vivo. Annu Rev Phys Chem.

[60] Soto-Sánchez C, Martínez-Navarrete G, Humphreys L, Puras G, Zarate J (2015). Enduring high-efficiency in vivo transfection of neurons with non-viral magnetoparticles in the rat visual cortex for optogenetic applications. Nanomedicine.

[61] Hashimoto M, Hisano Y (2011). Directional gene-transfer into the brain by an adenoviral vector tagged with magnetic nanoparticles. J Neurosci Methods.

[62] Zhou XF, Liu B, Yu XH, Zha X, Zhang XZ (2007). Using magnetic force to enhance immune response to DNA vaccine. Small.

[63] Almstätter I, Mykhaylyk O, Settles M, Altomonte J, Aichler M (2015). Characterization of magnetic viral complexes for targeted delivery in oncology. Theranostics.

[64] Lee JH, Kim JW, Cheon J (2013). Magnetic nanoparticles for multi-imaging and drug delivery. Mol Cells.

